# The prevalence of liver abnormalities in individuals with ZZ Alpha-1 Antitrypsin deficiency

**DOI:** 10.1186/1753-6561-9-S1-A40

**Published:** 2015-01-14

**Authors:** OF McElvaney, L Fee, C O’Connor, TP Carroll

**Affiliations:** 1 Department of Medicine, Beaumont Hospital, Dublin 9, Ireland

## Background

Alpha-1 antitrypsin deficiency (AATD) is a hereditary disorder defined by low plasma levels of alpha-1 antitrypsin (AAT). It is linked primarily with the development of lung, liver and skin disease. The most common abnormal variant of AAT is the ‘Z’ variant. It is the AATD type most associated with the development of liver disease. The aim of this project is to determine the prevalence of liver abnormalities in ZZ AATD individuals.

## Methods

The study cohort included 115 ZZ AATD patients. The study population was drawn from AATD patients attending the National AATD referral centre in Beaumont Hospital. The cohort is racially homogenous and the mean age is 52 ± 12 yrs (62 male and 53 female). All 115 patients provided serum samples which underwent confirmation of AATD status by phenotyping. Patients answered a standardized questionnaire about their social history and patient charts, abdominal ultrasound records, and liver function tests were reviewed to determine the incidence of liver abnormalities.

## Results

Of the 115 people with AATD in our study, 45 had liver function test abnormalities. There was no correlation between increasing age and liver function test abnormalities. Thirty of the study subjects had liver abnormalities on liver ultrasound, the majority having fatty infiltration. There was no difference in BMI in those with or without liver disease. Of the 115 studied 80 answered the personal and social history survey concerning alcohol consumption. In those with liver disease 21 out of 30 participated in the survey. There were no significant differences in alcohol consumption between those with liver disease and the general AATD population.

## Conclusions

In this study we found that liver disease is relatively common in the ZZ cohort, with over 25% having abnormal liver ultrasounds and over 35% having abnormal liver function tests. This is in keeping with other studies. We found no correlation between increasing age and abnormal liver function tests. We saw no link between alcohol consumption and abnormal liver ultrasounds. The frequency of fatty liver in the liver disease group raised the possibility that this was a result of increased obesity and not specifically AATD; however, there was no significant difference in BMI in this group compared to the overall ZZ population.
